# A new piece in the puzzle of the novel avian-origin influenza A (H7N9) virus

**DOI:** 10.1186/1745-6150-8-26

**Published:** 2013-10-26

**Authors:** Raphael Tze Chuen Lee, Vithiagaran Gunalan, Thanh Dac Van, Ly Thi Le, Frank Eisenhaber, Sebastian Maurer-Stroh

**Affiliations:** 1 Bioinformatics Institute (BII), Agency for Science Technology and Research (A*STAR), 30 Biopolis Street, #07-01, Matrix Building, 138671 Singapore, Singapore; 2 Life Science Laboratory, Institute of Computational Science and Technology, SBI Building, Quang Trung Software City, Tan Chanh Hiep Ward, District 12, Ho Chi Minh City, Vietnam; 3 School of Biotechnology, Ward 6, Linh Trung, Thu Duc District, International University – Vietnam National University, Ho Chi Minh City, Vietnam; 4 School of Computer Engineering (SCE), Nanyang Technological University (NTU), 50 Nanyang Drive, 637553 Singapore, Singapore; 5 Department of Biological Sciences (DBS), National University of Singapore (NUS), 8 Medical Drive 4, 117597 Singapore, Singapore; 6 School of Biological Sciences (SBS), Nanyang Technological University (NTU), 60 Nanyang Drive, 637551 Singapore, Singapore; 7 National Public Health Laboratory, Communicable Diseases Division Ministry of Health, Singapore, Singapore

**Keywords:** Avian influenza, Zoonotic infections, Phylogeny, Reassortment history

## Abstract

**Reviewers:**

This article was reviewed by Prof Xiufan Liu (nominated by Dr Purificacion Lopez-Garcia) and Prof Sandor Pongor.

Using phylogenetic analysis on newly available sequences, we characterize A/chicken/Jiangsu/RD5/2013(H10N9) as currently closest precursor strain for the NA segment in the novel avian-origin H7N9 virus responsible for an outbreak in China. We also show that the internal segments of this precursor strain are closely related to those of the presumed precursor for the HA segment, A/duck/Zhejiang/12/2011(H7N3), which indicates that the sources of both HA and NA donors for the reassortant virus are of regional and not migratory-bird origin and highlights the role of chicken already in the early reassortment events.

## Findings

**Figure 1:**
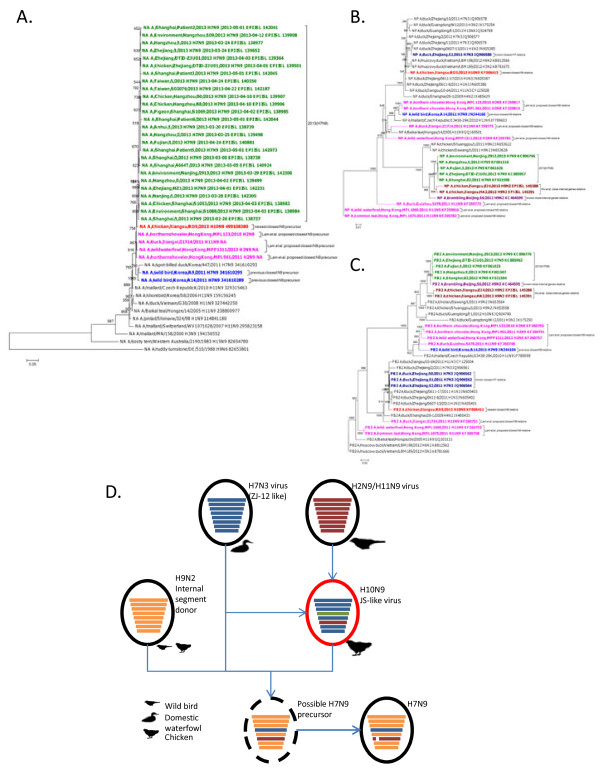
**A new JS5-like(H10N9) precursor is the closest NA segment relative to the 2013 H7N9 and its internal genes are closely related to the HA donor ZJ12-like(H7N3). (A)** Maximum likelihood phylogenetic tree of NA gene segments shows the NA from the novel avian-origin H7N9 virus (green) is more closely related to JS5-like (H10N9, red) than KO14-like (H7N9, blue) and precursors proposed by Lam *et al.* (pink). Methods: The H7N9 outbreak sequences were downloaded from GISAID’s EpiFlu™ database on June 13th 2013 (detailed acknowledgment in additional file 3) and complemented with best BLAST hits from Genbank as well as selected older representative strains as outgroup. The sequences were aligned with MAFFT using the L-INS-I parameters, the alignment was checked in Jalview and a nonredundant subset with less than 100% identity was retained. The phylogenetic tree was generated in PhyML with a gamma-distributed HKY85 substitution model. Positions with gaps and missing data were completely deleted, and 1000-replicate bootstrapping was performed; an average of these replicates is shown. **(B)**, **(C)** Phylogenetic analysis of internal segments amongst H7N9 strains and other closely related strains shows close identity between the ZJ12-like(H7N3, dark blue) HA donor and JS5-like(H10N9, red) NA donor. Maximum-likelihood trees using nucleotide sequences of **(B)** NP and (C) PB2 gene segments were generated as described before. Reassortant H7N9 strains (green), presumed BJ16-like internal segment donor (purple), previously postulated KO14-like (H7N9) NA donor (blue), newly proposed N9 relative from Lam *et al.* (pink) and internal gene relatives from Wu et al. (dark red) are also indicated. **(D)** The NA gene segment of the reassortant H7N9 virus derives from JS5-like (H10N9, red), the HA gene segment from ZJ12-like (H7N3, blue) and the internal segments derive from BJ16-like or ZJ04-like (H9N2, orange) strains.

The outbreak of a highly pathogenic novel avian-origin influenza A (H7N9) virus with multiple human fatalities earlier this year in the eastern provinces of China was a wake-up call for the pandemic potential of animal-hosted influenza strains and new subtypes outside of the typical surveillance focus. A remarkably efficient regional and international response resulted in a rapid genomic characterization including hypotheses on the evolutionary and reassortment history of this new virus [1-3]. The consensus picture that emerged in the first publications was that while the internal segments were of BJ16-like (H9N2) origin (e.g. A/brambling/Beijing/16/2012(H9N2)), the reassortant H7N9 virus received its hemagglutinin (HA) segment from ZJ12-like (H7N3) (e.g. A/duck/Zhejiang/12/2011(H7N3)) and its neuraminidase (NA) segment from KO14-like (H7N9) (e.g. A/wild bird/Korea/A14/2011(H7N9)) viruses, respectively. While this manuscript was submitted, two related new works were published and are relevant for our discussion. Lam *et al.*[4] reported sequence results from 388 new isolates sampled from domestic waterfowl and poultry in the region during the outbreak and Wu *et al.*[5] shed further light on different H9N2 sources of the internal gene segments. Lam *et al.* also highlight H11N9 and H2N9 precursors for the NA segment that were more closely related to the H7N9 causing recent human infections than the initially mentioned KO14-like (H7N9) viruses. However, we would like to draw attention to a new development which is the identification of a different closest precursor strain for the NA segment by Su *et al.*[6] which became available only after the first analyses were published but was not mentioned by Lam *et al.*. Contemporary to the H7N9 outbreak, A/chicken/Jiangsu/RD5/2013(H10N9) was collected in March 2013 from a live poultry market in Jiangsu and the genome submitted to Genbank [7] in May by Su *et al.* who also reported a quick characterization without phylogenetic analysis [6]. We have examined the detailed phylogenetic relationship of the NA of this new strain (hereafter referred to as JS5-like (H10N9)) using a maximum-likelihood tree (employing a gamma-distributed HKY85 substitution model [8] and 1000-replicate bootstrapping [9]) using MAFFT [10], Jalview [11] and PhyML [12] (Figure 1A) and confirmed that this JS5-like N9 NA was indeed more closely related to the H7N9 outbreak sequences than the previously postulated closest KO14-like (H7N9) ancestor and the H11N9 or H2N9 strains listed by Lam *et al.*. Notably, like KO14-like (H7N9) also JS5-like (H10N9) did not yet have the NA stalk deletion seen in the novel avian-origin influenza A (H7N9). In order to confirm the results of the maximum likelihood analysis, the same set of sequences was also analyzed using Neighbour-joining performed in MEGA5.2 [13-15] and Bayesian Markov Chain Monte Carlo analysis performed in BEAST [16] (see Additional file 1). The results from all analyses using different phylogenetic approaches identified the same JS5-like (H10N9) virus as being the closest ancestor to the reassortant NA, and hence served to validate the initial findings.

We next examined if other segments from this newer, closer NA precursor strain are closely related to other known H7N9 precursors. Phylogenetic analysis for the HA segment in JS5-like (H10N9) virus showed it to be more distant than the presumed ZJ12-like HA donor (see Additional file 2), which hence remains the most closely related putative precursor for HA. Interestingly, each of the 6 internal segments from A/chicken/Jiangsu/RD5/2013(H10N9) were shown to be closely related to the putative HA donor ZJ12-like (H7N3), differently from the KO14-like (H7N9) virus, with a minimum sequence identity of 98%. BLAST [17] searches for each of these internal segments from the previously postulated closest NA precursor A/wild bird/Korea/A14/2011(H7N9) did not return direct significant hits for the other known H7N9 precursors. Maximum likelihood phylogenetic analysis was performed for each of the JS5-like (H10N9) internal segments with KO14-like (H7N9), ZJ12-like (H7N3), BJ16-like (H9N2) and other related strains using PhyML, with a gamma-distributed HKY85 substitution model and with treatments similar to the analysis of other gene segments (complete deletion of positions with gaps and missing data, 1000-replicate bootstrapping). These analyses confirmed the initial BLAST findings (see Figure1B and C and Additional file 2). Given these interesting segment relations, we propose a revised model for the reassortment pathway leading to the genesis of the novel avian-origin H7N9 virus in Figure 1D.

In summary, we characterized the phylogeny of a new closest precursor for the NA segment and also found that the internal segments of this strain are closely related to those of the closest precursor of the HA segment. These two new findings are interesting for several reasons. First, the new related strain was found in the Jiangsu province which is much closer than the initially postulated NA donor and bordering the early affected Anhui, Shanghai and Zhejiang provinces. Second, the close relation between ZJ12-like (H7N3) and JS5-like (H10N9) through their internal segments narrows down the putative source region for this specific reassortment to the interface of Jiangsu and Zhejiang provinces which includes the Yangtze River delta and the city of Shanghai. Both of these factors add to the plausibility of the ZJ12-like (H7N3) and JS5-like (H10N9) viruses being connected more recently for possible co-infection and reassortment. Third, the JS5-like (H10N9) virus was found in chicken suggesting a possible regional presence of both HA and NA donors in poultry following the initial sporadic introduction from migrating wild birds which, fourth, also has implications on which animal hosts should be considered candidates for further surveillance and testing. Similarly, the origin of internal segments in the novel avian-origin influenza A (H7N9) virus has now been linked to a possible dual reassortment of different H9N2 lineages in chicken [4,5] and it seems that domestic poultry appears to be the key for the last and still missing steps completing the reassortments resulting in the novel avian-origin influenza A (H7N9) virus in Eastern China. Putting together the pieces of the reassortment puzzle of this virus allows tracing it back closer to its initial source and natural reservoir which is important for the control and the prevention of future similar outbreaks.

## Reviewers’ comments

### Reviewer number: 1

*Reviewer name:* Prof Xiufan Liu (nominated by Dr Purificacion Lopez-Garcia)

General comment:

Based on available sequences submitted to GenBank, the authors used different phylogenetic methods to analyze the NA origin of the novel avian-origin H7N9 virus causing recent human outbreak in China, and confirmed that A/chicken/Jiangsu/RD5/2013(H10N9) is the closest precursor strain for the NA segment in the H7N9 virus and its internal genes are closely related to those of the presumed precursor for the HA segment, A/duck/Zhejiang/12/2011(H7N3), first suggested by Su et al. The authors concluded that the sources of both HA and NA donors for the reassortant H7N9 virus are of regional and not migratory-bird origin. The manuscript is publishable after careful revision.

Specific comments:

1. According to the more recently published paper in Nature (Lam et al. The genesis and source of the H7N9 influenza viruses causing human infections in China, doi:10.1038/nature12515), the NA gene of H7N9 is more closely related to the NA gene from H11N9 and H2N9 viruses in migratory wild birds or H11N9 viruses in domestic ducks in China. Therefore, the phylogenetic analyses in Figure 1(A), S1-A and S1-B, S2-C should include N9 sequences from these strains to determine if A/chicken/Jiangsu/RD5/2013(H10N9) is the closest precursor strain.

2. In Figure 1(D), the authors propose a revised model for the reassortment pathway leading to the genesis of the novel avian-origin H7N9 virus: the reassortant H7N9 viruses of avian origin derive their internal genes from BJ16-like (H9N2) virus of wild bird. However, more recently, reports indicated that the novel H7N9 viruses with diverse genotypes were generated by sequential reassortments involving distinct H9N2 donor viruses in different hosts (Lam et al., The genesis and source of the H7N9 influenza viruses causing human infections in China. Nature, 2013, doi:10.1038/nature12515; Wu et al. Sequential Reassortments Underlie Diverse H7N9 genotypes in China. Cell Host and Microbe, 2013. http://dx.doi.org/10.1016/j.chom.2013.09.001). Therefore, Figure 1(D) should be revised.

Author’s Reply: Many thanks for pointing to these papers which have only been published after initial submission of our manuscript. We totally agree that in order to provide the best current state-of-the-art view of the H7N9 origins the newly reported sequences should be included in our analysis and we therefore did the additional work including sequences from both papers. It turns out that the Su et al. strain A/chicken/Jiangsu/RD5/2013(H10N9) we discuss here is still the closest NA precursor and the Lam et al. H11N9 and H2N9 strains are in-between the KO14-like (H7N9) and JS5-like (H10N9). Related to comment 2, while the discussion on H9N2-derived internal segments is not the main focus of this work, we are now citing Wu et al. for details on this and have also revised Figure 1D to account for the new findings of sequential reassortments from different hosts.

### Reviewer number: 2

*Reviewer name:* Prof Sandor Pongor

This work by Lee et al. attempts to piece together influenza strains that can explain emergence of the novel H7N9 in China. This is very important topic at the moment and the findings and conclusions of this manuscript contribute to our current understanding of the matter. While this manuscript was under submission, a new work was published in Nature that addresses the genesis and source of the H7N9 influenza viruses causing human infections in China (Lam et al. Nature. 2013 Aug 21. doi:10.1038/nature12515). At first glance, this new paper does not seem to include the strain discussed by Lee et al. The authors are encouraged to compare their findings with those of Lam et al. at least in a dedicated paragraph and discuss where their strain fits into the overall picture.

Author’s Reply: Many thanks for the comment. Also Reviewer 1, who is one of the submitters of the Su et al. strain, pointed this out and our reply is hence the same. In short, in order to provide the best current state-of-the-art view of the H7N9 origins we included the newly reported sequences in our analysis. It turns out that the Su et al. strain A/chicken/Jiangsu/RD5/2013(H10N9) we discuss here is still the closest NA precursor and the Lam et al. H11N9 and H2N9 strains are in-between the KO14-like (H7N9) and JS5-like (H10N9).

## Competing interests

All authors declare that they have no competing interests.

## Supplementary Material

Additional file 1 **Phylogenetic trees of NA gene segments validate the finding that the NA gene segment from the novel avian-origin H7N9 virus (green) is more closely related to JS5-like (H10N9, red) than KO14-like (H7N9, blue) and closely related strains.** (S1-A) Neighbour joining (Maximum Composite Likelihood model with uniform site substitution rates) tree generated in MEGA5.2 with 1000 bootstrap replicates and complete deletion of positions with gaps and missing data. Presumed BJ16-like internal segment donor (purple) and newly proposed closest N9 relative from Lam *et al.* (pink) are also indicated. (S1-B) Bayesian Markov Chain Monte Carlo tree (gamma distributed HKY85 nucleotide substitution model) generated using BEAST with a strict clock model for uniform rates across branches. The MCMC chain length was 10000000 logged every 1000 steps and the first 10000 trees were removed as burn-in. Click here for file

Additional file 2 **The closest ZJ12-like(H7N3) relative is genetically similar to the new closest N9 JS5-like(H10N9) relative.** Phylogenetic trees of six genes PB1 (S2-A), PA (S2-B), NA (S2-C), MP (S2-D), NS (S2-E) and HA (S2-F) show the closest H7 relative A/duck/Zhejiang/12/2011(H7N3, dark blue) is genetically similar to the new closest N9 relative A/chicken/Jiangsu/RD5/2013(H10N9, red) in all internal gene segments (see Figure 1B & C in main text for PB2 and NP trees). PHYML was used to generate the maximum likelihood trees with a gamma-distributed HKY85 substitution model and 1000-replicate bootstrap testing for each tree. Reassortant H7N9 strains (green), presumed BJ16-like internal segment donor (purple), previously postulated KO14-like (H7N9) NA donor (blue), newly proposed N9 relative from Lam *et al.*[4] (pink) and internal genes relative from Wu *et al*. [5] (dark red) are also indicated. Click here for file

Additional file 3 Acknowledgement List of GISAID contributors. Click here for file
