# Supplementary figures and images for: A new piece in the puzzle of the novel avian-origin influenza A (H7N9) virus
Source: Biol Direct. 2013 Oct 26;8:26. doi: 10.1186/1745-6150-8-26 (PMC4016609; doi:10.1186/1745-6150-8-26)

S1-A

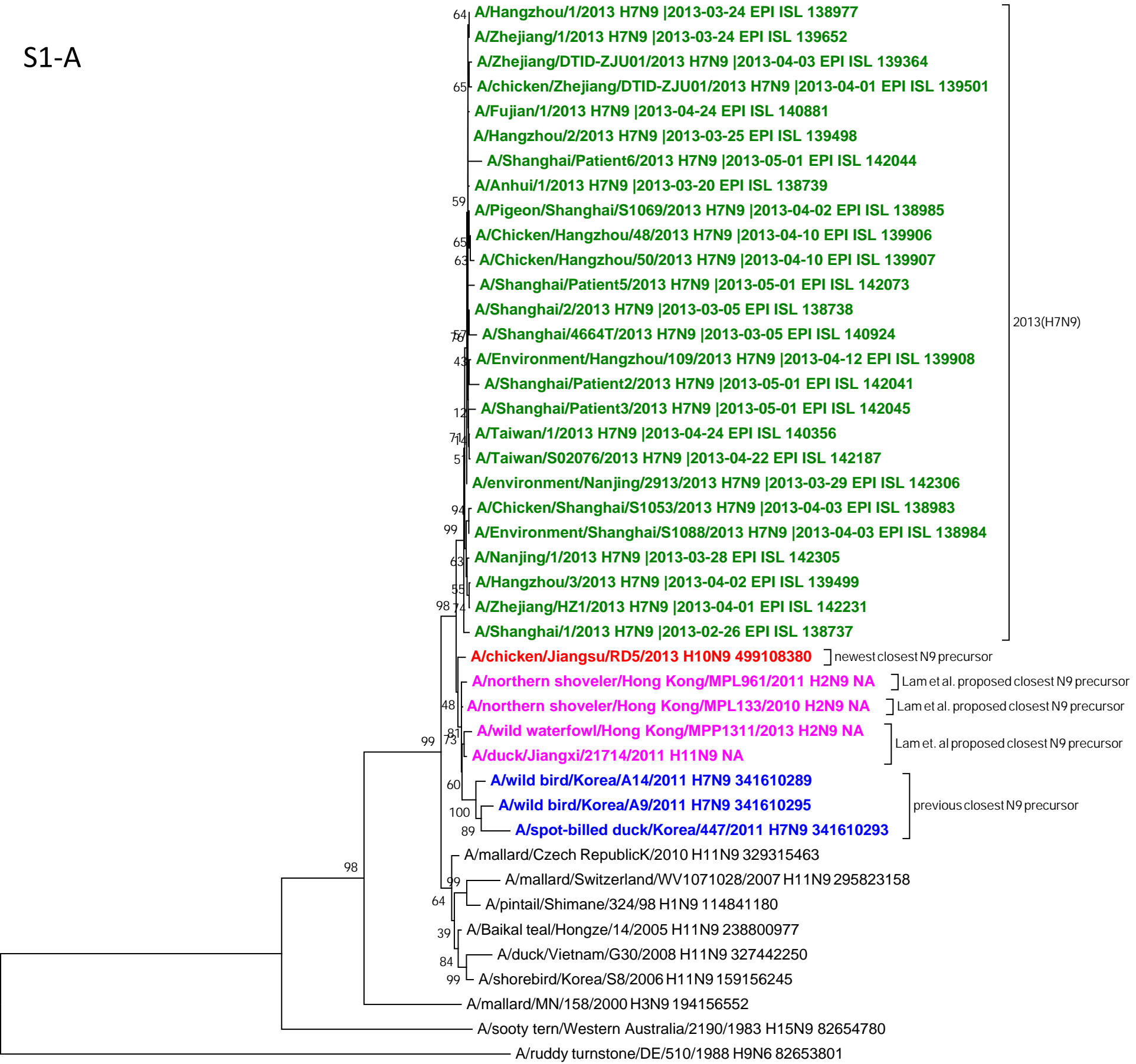

0.05

S1-B

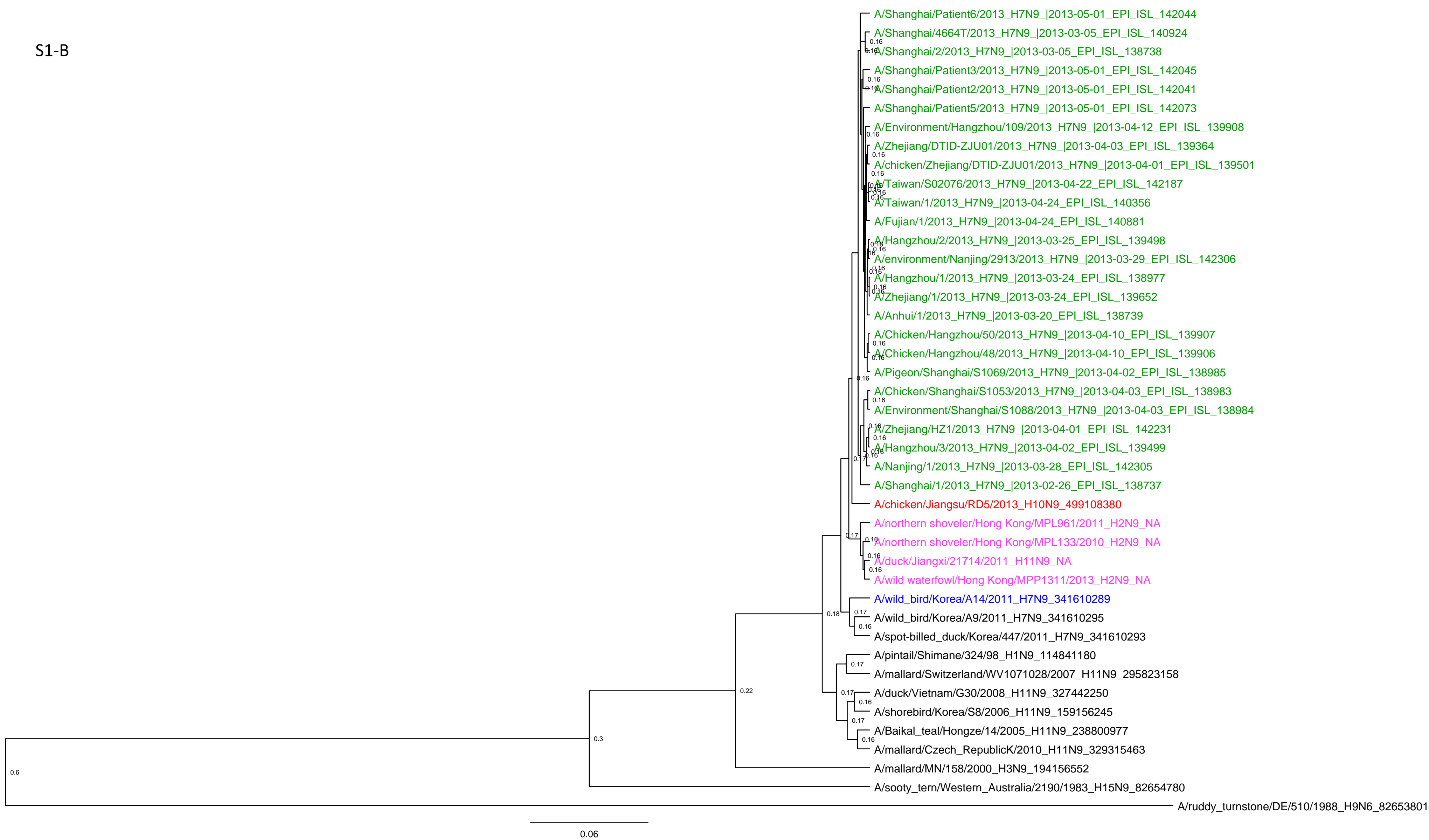

Supplement: Additional file 1 — Phylogenetic trees of NA gene segments validate the finding that the NA gene segment from the novel avian-origin H7N9 virus (green) is more closely related to JS5-like (H10N9, red) than KO14-like (H7N9, blue) and closely related strains. (S1-A) Neighbour joining (Maximum Composite Likelihood model with uniform site substitution rates) tree generated in MEGA5.2 with 1000 bootstrap replicates and complete deletion of positions with gaps and missing data. Presumed BJ16-like internal segment donor (purple) and newly proposed closest N9 relative from Lam et al. (pink) are also indicated. (S1-B) Bayesian Markov Chain Monte Carlo tree (gamma distributed HKY85 nucleotide substitution model) generated using BEAST with a strict clock model for uniform rates across branches. The MCMC chain length was 10000000 logged every 1000 steps and the first 10000 trees were removed as burn-in. [file 1745-6150-8-26-S1.pdf]

S2-A

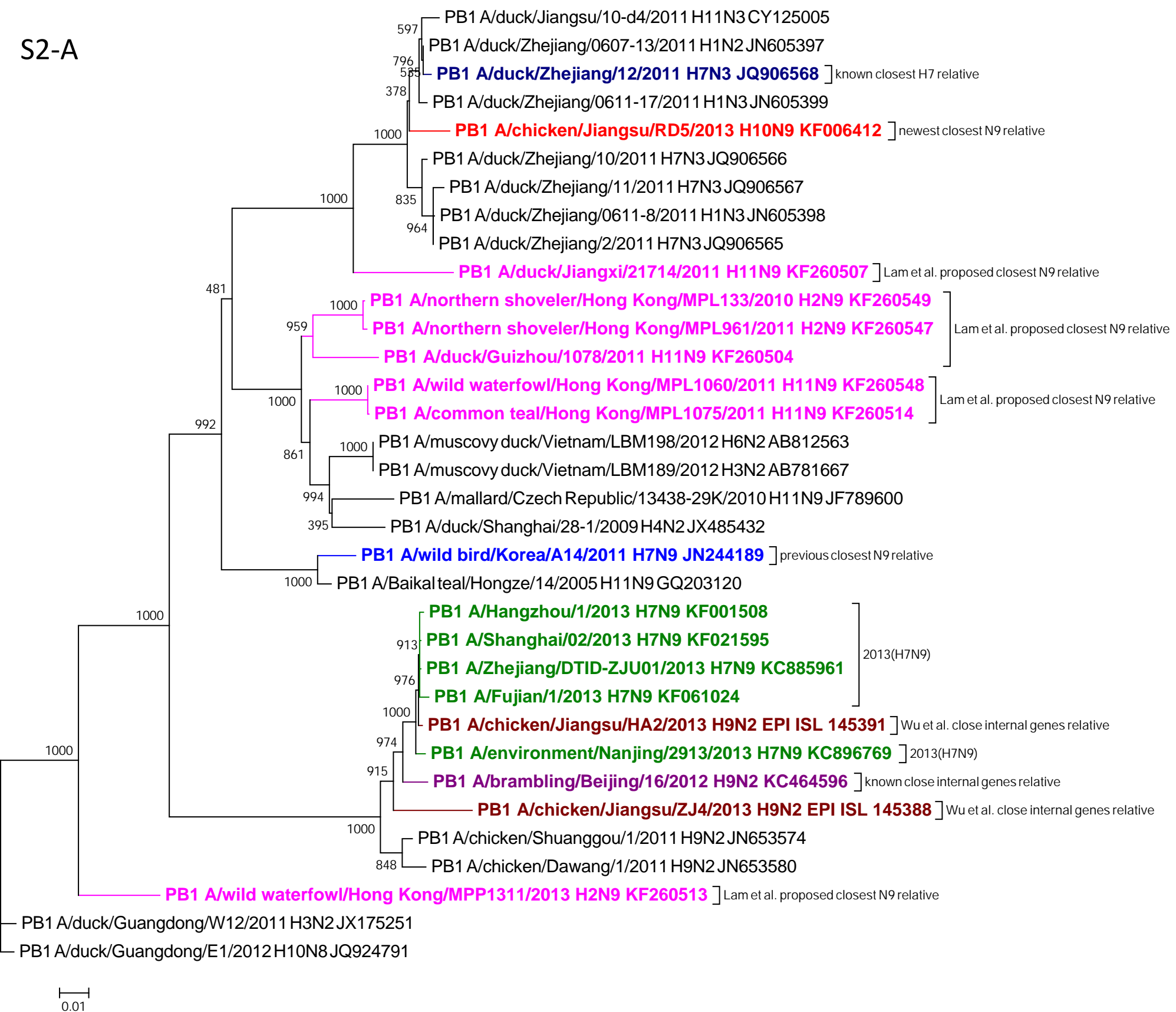

S2-B

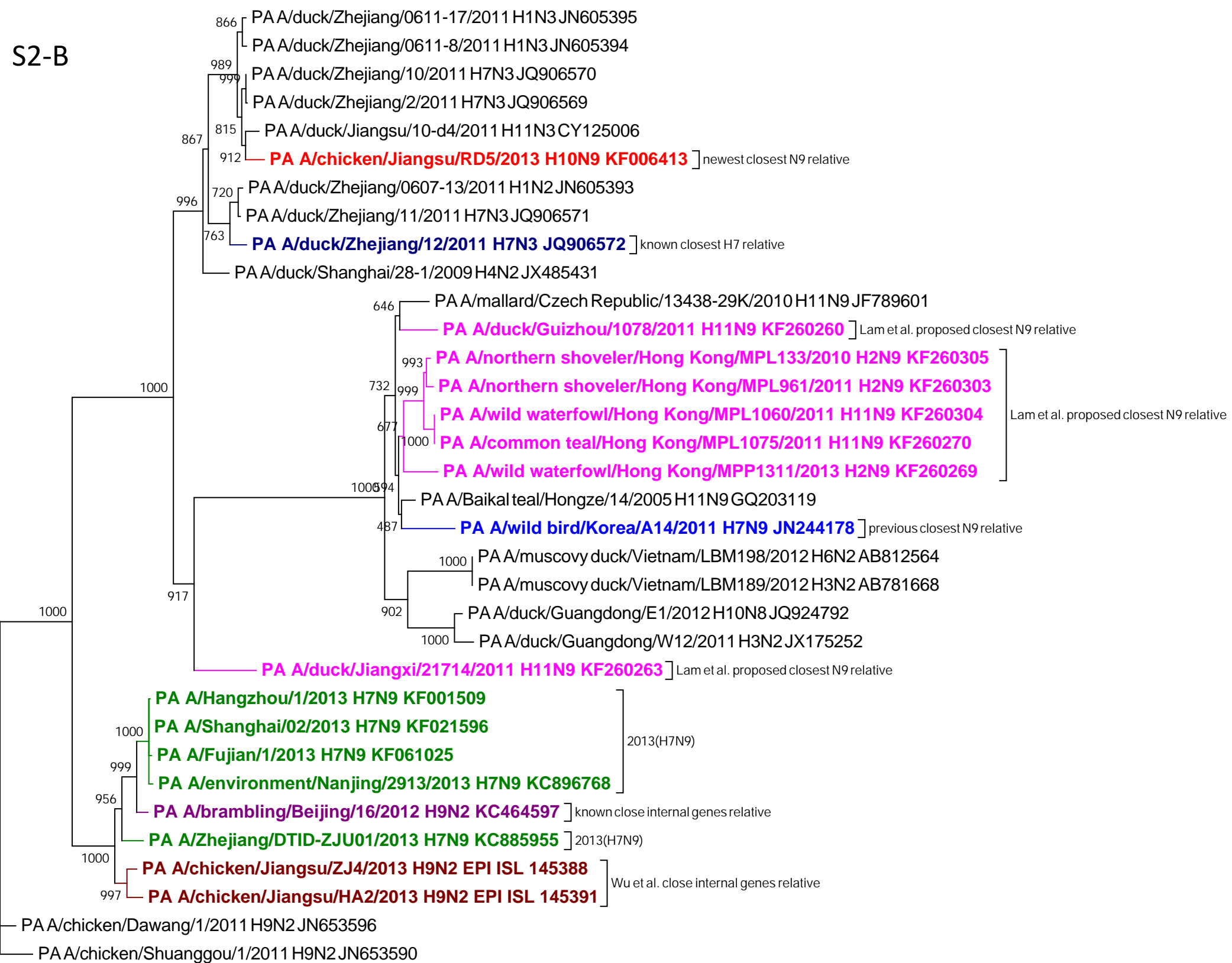

0.01

S2-C

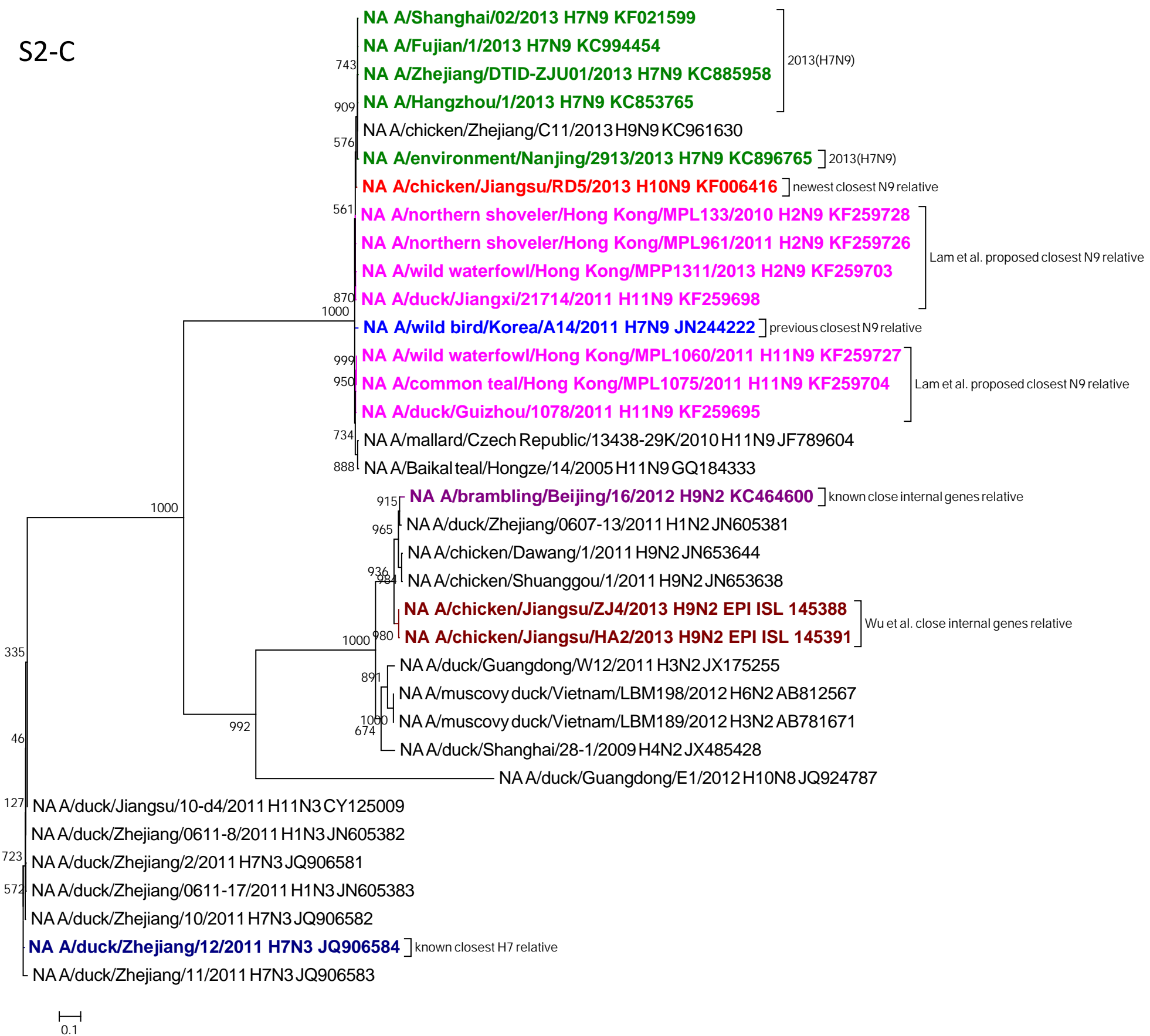

S2-D

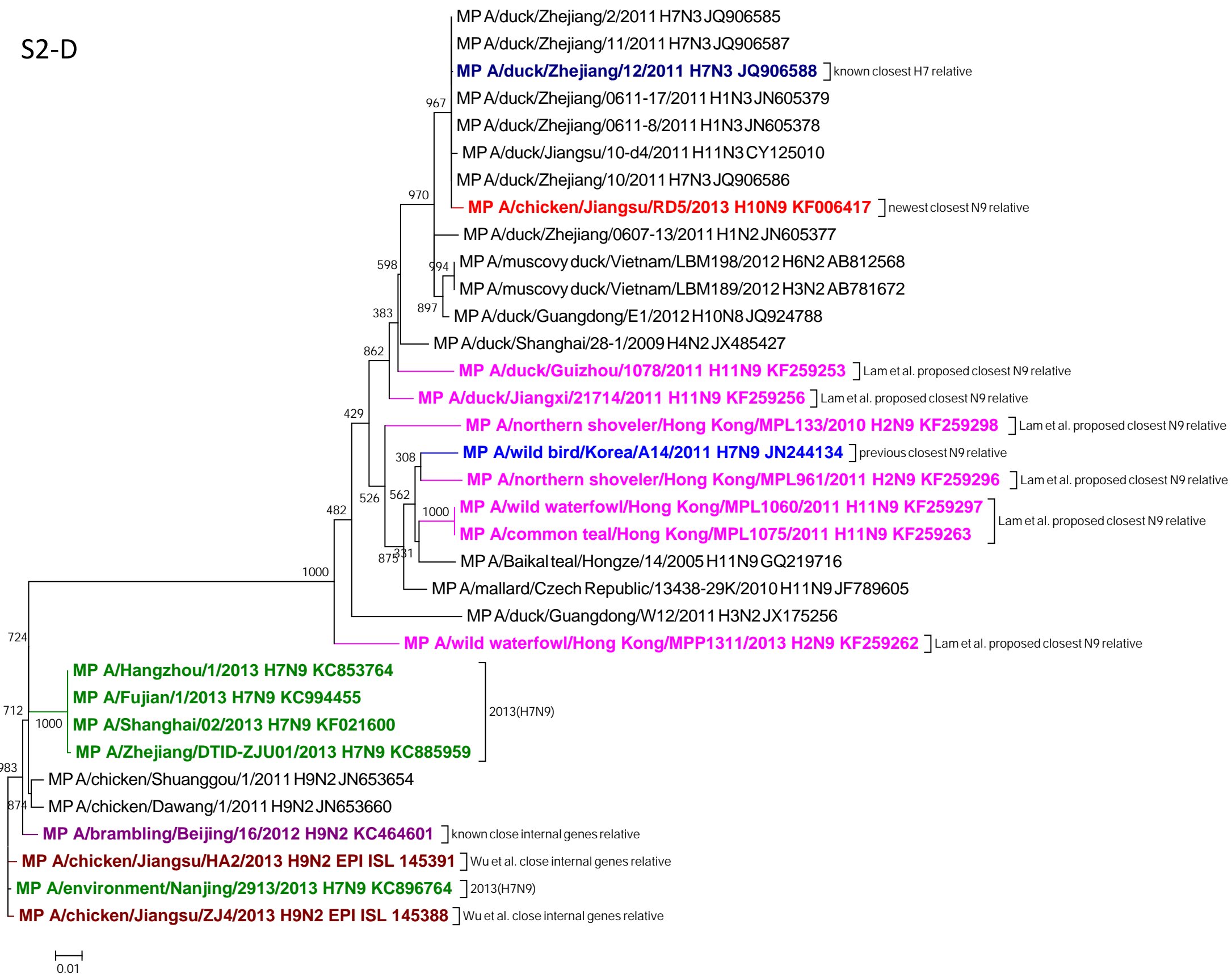

S2-E

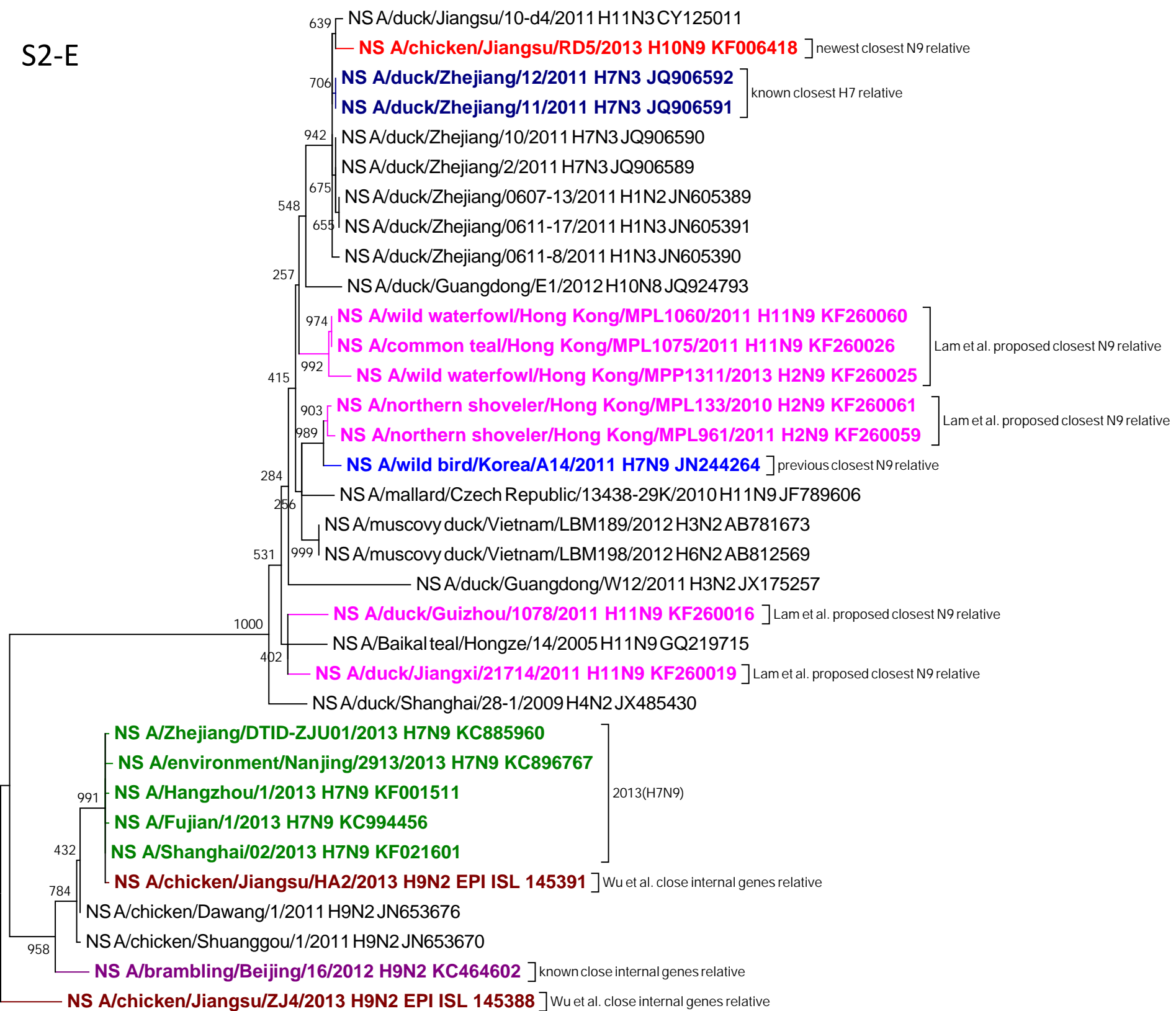

0.01

S2-F

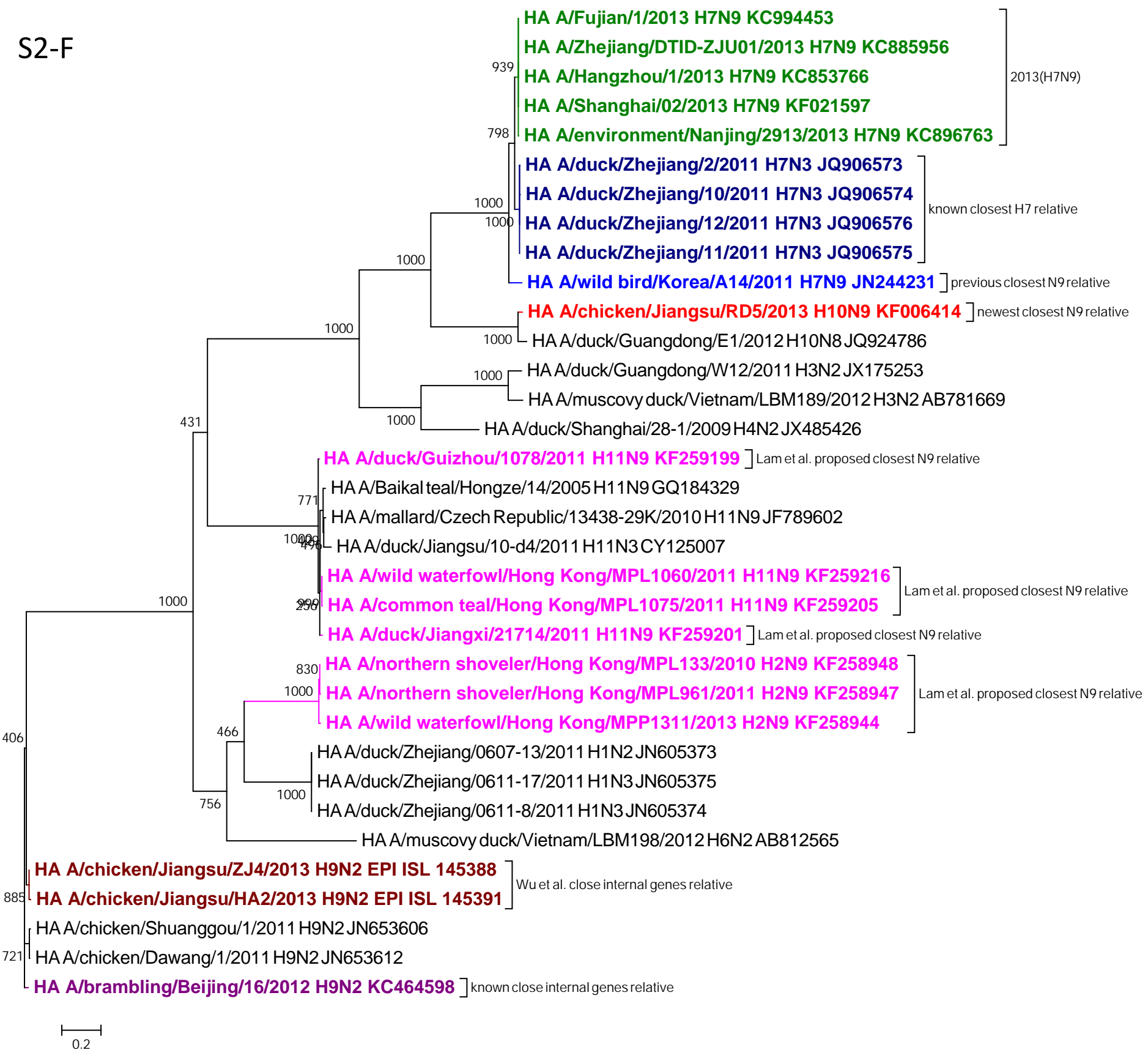

Supplement: Additional file 2 — The closest ZJ12-like(H7N3) relative is genetically similar to the new closest N9 JS5-like(H10N9) relative. Phylogenetic trees of six genes PB1 (S2-A), PA (S2-B), NA (S2-C), MP (S2-D), NS (S2-E) and HA (S2-F) show the closest H7 relative A/duck/Zhejiang/12/2011(H7N3, dark blue) is genetically similar to the new closest N9 relative A/chicken/Jiangsu/RD5/2013(H10N9, red) in all internal gene segments (see Figure 1B & C in main text for PB2 and NP trees). PHYML was used to generate the maximum likelihood trees with a gamma-distributed HKY85 substitution model and 1000-replicate bootstrap testing for each tree. Reassortant H7N9 strains (green), presumed BJ16-like internal segment donor (purple), previously postulated KO14-like (H7N9) NA donor (blue), newly proposed N9 relative from Lam et al.[4] (pink) and internal genes relative from Wu et al. [5] (dark red) are also indicated. [file 1745-6150-8-26-S2.pdf]
