# Supplementary material for: A new piece in the puzzle of the novel avian-origin influenza A (H7N9) virus
Source: Biol Direct. 2013 Oct 26;8:26. doi: 10.1186/1745-6150-8-26 (PMC4016609; doi:10.1186/1745-6150-8-26)
Supplement: Additional file 3 — Acknowledgement List of GISAID contributors. [file 1745-6150-8-26-S3.pdf]

We acknowledge the authors, originating and submitting laboratories of the sequences from GISAID's EpiFlu™ Database on which this research is based. The list is detailed below.

All submitters of data may be contacted directly via the GISAID website *www.gisaid.org*

Segment ID	Segment	Country	Collection date	Isolate name	Originating Lab	Submitting Lab	Authors
EPI440700	NA	China	2013-Apr-02	A/Pigeon/Shanghai/S1069/2013	Harbin Veterinary Research Institute	Harbin Veterinary Research Institute	
EPI453611	NA	China	2013-Mar-29	A/environment/Nanjing/2913/2013		Other Database Import	Bao,C.; Cui,L.; Zhou,M.; Guo,X.; Hong,L.; Gao,G.F.; Wang,H.; Qi,X.; Ge,Y.; Zhao,K.; Li,W.; Tang,F.; Shi,Z.; Li,Y.
EPI440692	NA	China	2013-Apr-03	A/Environment/Shanghai/S1088/2013	Harbin Veterinary Research Institute	Harbin Veterinary Research Institute	
EPI443677	NA	China	2013-Apr-12	A/Environment/Hangzhou/109/2013	Hangzhou Center for Disease Control and Prevention	Hangzhou Center for Disease Control and Prevention	Li,J;Yu,XF;Pu,XY;Xie,L;Sun,YX;Xiao,HX;Wang,FJ;Din,H;Wu,Y;Liu,D;Zhao,GQ;Liu,J;Pan,JC
EPI445914	NA	Taiwan	2013-Apr-24	A/Taiwan/1/2013	National Influenza Center, Centers for Disease Control	Taiwan CDC	Ji-Rong, Yang; Ming-Tsan, Liu; Ho-Sheng, Wu
EPI451333	NA	China	2013-May-01	A/Shanghai/Patient5/2013	Shanghai public health clinical center	Institute Pasteur of Shanghai, CAS	
EPI451325	NA	China	2013-May-01	A/Shanghai/Patient3/2013	Shanghai public health clinical center	Institute Pasteur of Shanghai, CAS	
EPI451274	NA	China	2013-May-01	A/Shanghai/Patient6/2013	Shanghai public health clinical center	Institute Pasteur of Shanghai, CAS	
EPI451255	NA	China	2013-May-01	A/Shanghai/Patient2/2013	Shanghai public health clinical center	Institute Pasteur of Shanghai, CAS	
EPI443036	NA	China	2013-Mar-24	A/Zhejiang/1/2013	Zhejiang Provincial Center for Disease Control and Prevention	Zhejiang Provincial Center for Disease Control and Prevention	Sun, Y; Zhang, YJ
EPI442714	NA	China	2013-Apr-02	A/Hangzhou/3/2013	Hangzhou Center for Disease Control and Prevention	Hangzhou Center for Disease Control and Prevention	Jing-Cao, Pan
EPI442711	NA	China	2013-Mar-25	A/Hangzhou/2/2013	Hangzhou Center for Disease Control and Prevention	Hangzhou Center for Disease Control and Prevention	Jing-Cao, Pan
EPI440096	NA	China	2013-Mar-24	A/Hangzhou/1/2013	Hangzhou Center for Disease Control and Prevention	Hangzhou Center for Disease Control and Prevention	Li,J; Sun,YX; Pu,XY; Yu,XF; Chen,QJ; Kou,Y; Ding,SJ; Zhou,YY; Pan,JC
EPI453477	NA	China	2013-Apr-01	A/Zhejiang/HZ1/2013		Other Database Import	Wu,H.; Wu,N.; Guo,C.
EPI446747	NA	China	2013-Apr-24	A/Fujian/1/2013		Other Database Import	Weng,Y.; Zhang,Y.; Xie,J.; Huang,M.; Chen,W.; He,W.; Wu,B.; Wang,J.; Yang,S.; Zheng,K.; Yan,Y.
EPI441797	NA	China	2013-Apr-03	A/Zhejiang/DTID-ZJU01/2013		Other Database Import	Chen,H.-L.; Yuen,K.-Y.; Chen,Y.; Wu,N.P.; Yang,S.G.; Wo,J.E.; Yao,H.P.; Liang,W.F.; Cui,D.W.; Wu,H.B.; Li,L.J.
EPI453606	NA	China	2013-Mar-28	A/Nanjing/1/2013		Other Database Import	Bao,C.; Cui,L.; Zhou,M.; Guo,X.; Hong,L.; Gao,G.F.; Wang,H.; Qi,X.; Ge,Y.; Zhao,K.; Li,W.; Tang,F.; Shi,Z.; Li,Y.
EPI452262	NA	Taiwan	2013-Apr-22	A/Taiwan/S02076/2013		Other Database Import	Chang,S.-C.; Chang,S.-Y.
EPI446965	NA	China	2013-Mar-05	A/Shanghai/4664T/2013		Other Database Import	Hu,Y.
EPI439509	NA	China	2013-Mar-20	A/Anhui/1/2013		WHO Chinese National Influenza Center	
EPI439500	NA	China	2013-Mar-05	A/Shanghai/2/2013		WHO Chinese National Influenza Center	
EPI439487	NA	China	2013-Feb-26	A/Shanghai/1/2013		WHO Chinese National Influenza Center	
EPI443669	NA	China	2013-Apr-10	A/Chicken/Hangzhou/50/2013	Hangzhou Center for Disease Control and Prevention	Hangzhou Center for Disease Control and Prevention	Li,J;Yu,XF;Pu,XY;Xie,L;Sun,YX;Xiao,HX;Wang,FJ;Din,H;Wu,Y;Liu,D;Zhao,GQ;Liu,J;Pan,JC
EPI443661	NA	China	2013-Apr-10	A/Chicken/Hangzhou/48/2013	Hangzhou Center for Disease Control and Prevention	Hangzhou Center for Disease Control and Prevention	Li,J;Yu,XF;Pu,XY;Xie,L;Sun,YX;Xiao,HX;Wang,FJ;Din,H;Wu,Y;Liu,D;Zhao,GQ;Liu,J;Pan,JC
EPI442723	NA	China	2013-Apr-01	A/chicken/Zhejiang/DTID-ZJU01/2013		Other Database Import	Wu,H.; Wu,N.; Yao,H.; Chen,H.; Song,W.; Wang,P.; Zheng,M.; Lau,S.; Li,L.
EPI440684	NA	China	2013-Apr-03	A/Chicken/Shanghai/S1053/2013	Harbin Veterinary Research Institute	Harbin Veterinary Research Institute	
EPI466481	NA	China	2013-Apr-05	A/chicken/Jiangsu/HA2/2013	Yangzhou University	Chinese Academy of Sciences	Daxin, P
EPI466457	NA	China	2013-Jan-23	A/chicken/Jiangsu/ZJ4/2013	Yangzhou University	Chinese Academy of Sciences	Daxin, P
